# Reply to: Exegesis on using a customized GPT to provide guideline-based recommendations for management of pancreatic cystic lesions

**DOI:** 10.1055/a-2605-1278

**Published:** 2025-06-17

**Authors:** Yuri Gorelik

**Affiliations:** 1 58878 Department of Internal Medicine D, Gastroenterology, Rambam Health Care Campus, Haifa, Israel



I have carefully read the letter to the editor entitled "Exegesis on using a customized GPT to provide guideline-based recommendations for the management of pancreatic cystic lesions" regarding our manuscript entitled "Using a customized GPT to provide guideline-based recommendations for management of pancreatic cystic lesions" 1 . I would like to sincerely thank Prof. Sengul for his insightful comments. I fully agree that the concerns raised—limited sample size and the analysis of variability—are critical areas to address in future research involving LLMs.

Our study was a pilot aimed at evaluating the potential of LLMs to navigate clinical scenarios in which guidelines differ, as evidenced by varying expert responses and guideline discrepancies. The findings demonstrated that GPT is capable of integrating diverse data and providing accurate recommendations.

To address these important points, we are currently conducting larger-scale studies using real-world data, thereby significantly increasing the sample size. In addition, newer LLMs now possess advanced reasoning capabilities (reasoning models), and their reasoning process can be reviewed and studied. This functionality allows for deeper exploration, improved inquiry, and optimization of model performance and clinical utility.

We anticipate that these efforts will enhance our understanding of the potential and capabilities of LLMs in providing decision support especially, in highly discrepant fields.

**Publication note**

Letters to the editor do not necessarily represent the opinion of the editor or publisher. The editor and publisher reserve the right to not publish letters to the editor, or to publish them abbreviated or in extracts.
